# Metabolomic changes in patients with chronic obstructive pulmonary disease with abnormal Savda syndrome

**DOI:** 10.3892/etm.2014.2085

**Published:** 2014-11-24

**Authors:** WEI-FANG XU, HALMURAT UPUR, YU-HUA WU, BATUR MAMTIMIN, JIAN YANG, YONG-JUAN GA, LI YOU

**Affiliations:** 1 Department of Respiratory Internal Medicine, The Affiliated Chinese Medicine Hospital of Xinjiang Medical University, Urumqi, Xinjiang 830000, P.R. China; 2 Department of Traditional Uyghur Medicine, Xinjiang Medical University, Urumqi, Xinjiang 830054, P.R. China; 3 Kashgar People’s Hospital of Xinjiang Uyghur Autonomous Region, Kashgar, Xinjiang 840000, P.R. China; 4 MRI Analysis Center of the College of Pharmacy, Xinjiang Medical University, Urumqi, Xinjiang 830011, P.R. China

**Keywords:** biomarkers, Traditional Uyghur Medicine, chronic obstructive pulmonary disease, abnormal Savda syndrome

## Abstract

The aim of this study was to determine the metabolic biomarkers for abnormal Savda syndrome in patients with chronic obstructive pulmonary disease (COPD). Based on Traditional Uyghur Medicine (TUM) theory, a total of 103 patients with COPD were classified into abnormal Savda and non-abnormal Savda syndrome groups and 52 healthy volunteers acted as the control group. Blood samples from the three groups were analyzed using nuclear magnetic resonance (NMR) spectroscopy combined with orthogonal projection to latent structure-discriminant analysis. NMR tests showed that the regional distributions of the patients with COPD with abnormal Savda syndrome, those with non-abnormal Savda syndrome and the control group were completely separate (P>0.05). The patients with COPD with abnormal Savda syndrome exhibited relatively low levels of amino acids, glycoproteins and unsaturated lipids (P<0.05) but significantly higher levels of lactic acid, carnitine, acetone and acetoacetate (P<0.05) compared with the healthy controls. Abnormal Savda syndrome was one of the main types of syndrome among the patients with COPD; increased age, a longer duration of illness and a higher disease severity were characteristic of this type of syndrome. In addition, the present study provided biochemical evidence for the TUM theory-based classification of patients with COPD; these biomarkers can be used in the clinic for the diagnosis of COPD with abnormal Savda syndrome. The study also demonstrated that the plasma metabolic disorder in patients with COPD with abnormal Savda syndrome was more serious than that in the control and COPD with non-abnormal Savda syndrome groups. The plasma metabolic disorder was also associated with a low immune function of the body and endocrine and energy metabolism disorders.

## Introduction

Chronic obstructive pulmonary disease (COPD), which is characterized by a partially irreversible and progressive airflow limitation, is associated with an abnormal inflammatory response of the lungs to harmful gases, such as cigarette smoke, and harmful particles (1). COPD primarily involves the lungs, but it can also cause a systemic inflammatory response, seriously affecting the patients’ ability to work and quality of life. COPD is one of the major diseases posing a threat to public health (2); however, despite the increasing prevalence of the disease, treatment with conventional Western medicine is expensive and can cause serious side effects. Furthermore, the optimal maintenance therapy has not yet been determined due to conflicting results regarding the efficacy and safety of the available medications (3).

Standardized research on the traditional medicine-based prevention and treatment of complex diseases, such as COPD, should be considered to provide an alternative option to Western medicine. Traditional Uyghur Medicine (TUM) has unique theories relevant to COPD, as well as formulae that may exert curative effects in patients with the disease.

One basic theory of TUM is that of body fluid (Hilit); Hilit is regarded as basic matter during physiological activity, which is produced in the liver as a result of a variety of foods, and is thought to provide the energy for other organs. According to the body fluid theory, Hilit includes Savda, Belghem, Sapra and Kan, which circulate in the body and are believed to maintain a corresponding balance. It is also believed that an abnormal change in Hilit leads to disease, a state that is termed abnormal Hilit; this is divided into abnormal Savda, Belghem, Sapra and Kan. Amongst them, abnormal Savda is particularly significant due to its links with diseases, including neoplasm, diabetes mellitus, hypertension and asthma COPD.

According to TUM theory, abnormal changes in body fluids, including abnormal Savda, Belghem, Sapra and Kan, are the common underlying features of complex diseases, particularly abnormal Savda (4,5). Abnormal Savda syndrome may be the most common type of syndrome in patients with COPD. The concept of the syndromes in TUM includes numerous aspects of a disease that affects multiple organs and systems (4,6) Using metabolomics as an approach may be beneficial in classifying the different syndromes and their corresponding metabolic networks. Therefore, the aim of this study was to conduct a plasma metabolomic analysis to determine the characteristics of patients with COPD with abnormal Savda syndrome using nuclear magnetic resonance (NMR) spectroscopy technology.

## Materials and methods

### Patient classification

A total of 103 male and female patients with COPD were enrolled in the study from the Affiliated Chinese Medicine Hospital of Xinjiang Medical University (Urumqi, China). The patients exhibited three disease severities, mild (n=15), moderate (n=38) and severe (n=50), and were aged between 40 and 80 years. According to TUM theory, the patients were classified into two groups: Abnormal Savda syndrome (n=48, including 3 mild, 11 moderate and 34 severe) and non-abnormal Savda syndrome (n=55, including 12 mild, 27 moderate and 16 severe; all the patients in this group exhibited abnormal Belghem, Sapra and Kan). A total of 52 healthy volunteers were assigned to the control group. The study protocol was approved by the Ethics Committee of Xinjiang Medical University, and all subjects provided written informed consent.

### Sample preparation

Blood samples (3 ml/individual) from the patients and healthy volunteers were collected in the morning prior to breakfast and centrifuged at 3,800 × g for 10 min. Following separation, the plasma was stored at −80°C. Prior to conducting the NMR experiments, the samples were thawed at room temperature. Each sample (200 μl) was then mixed with 400 μl phosphate-buffered saline and centrifuged at 4°C and 12,000 × g for 10 min. A 550-μl sample of the supernatant was added to an NMR test tube (5 mm in diameter) for further analysis.

### Acquisition of spectral data

^1^H NMR spectra from the samples were recorded on a spectrometer (Inova 600, Varian Medical Systems, Palo Alto, CA, USA) operated at 599.95 MHz, employing a standard one-dimensional NMR experiment with the Carr-Purcell-Meiboom-Gill technique; the relaxation delay was set to 2 sec and the acquisition time was 1.64 sec. For each spectrum, a total of 128 transients were collected into 32,768 data-points with a spectral width of 20 ppm at 298 Kelvin. All free induction decays were multiplied by an exponential function equivalent to a 0.3 Hz line-broadening factor prior to Fourier transformation. For assignment purposes, standard two-dimensional (2D) NMR experiments were conducted on selected samples, including correlation spectroscopy (COSY), total correlation spectroscopy (TOCSY) and J-Resolved spectroscopy (J-Res) NMR spectra.

### Data processing and analysis

Fourier-transformed ^1^H NMR spectra were corrected for phase and baseline distortions. The chemical shifts of the spectra were referenced to the anomeric proton of the α-glucose signal at 5.233 ppm, and the range of 8.5-0.5 ppm was divided into 2,834 integrated areas. The integral values between 5.23 and 4.66 ppm were excluded from the analysis as they contained water resonances. The spectra were normalized over the total sum of the remaining spectral area.

Analyses of the integral results were performed with orthogonal projection to latent structure-discriminate analysis (OPLS-DA). OPLS-DA, including an orthogonal signal correction (OSC) in the partial least squares DA, was used for the extraction of COPD-related biomarkers by removing the influence of systematic variations not associated with COPD. The OSC was capable of eliminating the influence of dietary, age, gender and environmental factors and of decreasing the heterogeneity of samples, a particularly useful technique for clinical investigations (7,8).

In this study, NMR data were analyzed with OPLS-DA in accordance with the methods outlined in previous studies (9,10), and were processed automatically prior to analysis (unit variance scaling). The formula utilized was as follows: X × ij = (Xij-Mj)/Sj (Varian units using the Variance scaling). In this formula, Sj was the variance of variable j. After the transformation, the proportion of metabolites of the higher contents was decreased, and the proportion of high and low levels of metabolites could be discriminated; thus, the low signal levels of metabolites could be improved due to the equally strong and weak signal contribution to the model that truly contributed to the distinction between the groups of metabolites. R^2^X, R^2^Y and Q^2^ represented the established quality evaluation indices of the model. For each model, the values of R^2^X and R^2^Y described the variance of X and Y, respectively, while Q^2^ represented the cross-validation parameter and indicated the predictability of the model. Thus, the authenticity of the results could be determined. With regard to the cross-validation results, when Q^2^>0.4, the prediction results were accepted (11) and when Q^2^>0.9, the prediction results of the model were considered to be the most reliable (12).

## Results

### ^1^H NMR spectra of plasma samples

**Figure 1:**
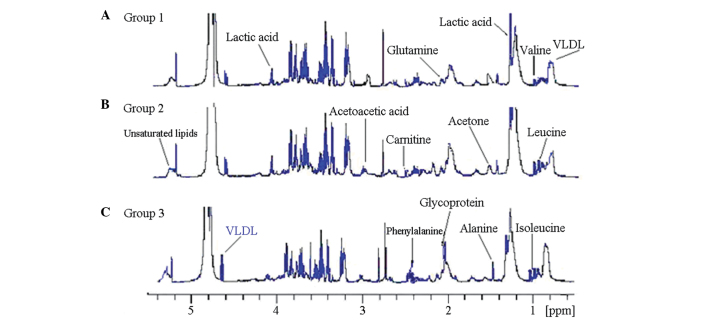
Typical ^1^H nuclear magnetic resonance 500 MHz spectra of plasma from patients with chronic obstructive pulmonary disease with (A) abnormal Savda syndrome (group 1) and (B) non-abnormal Savda syndrome (group 2); (C) healthy controls (group 3).

Fig. 1 shows the original record of the ^1^H NMR spectra of the plasma from the patients with COPD and healthy controls. The metabolomics-related ^1^H NMR chemical shift spectrum can be found in the Human Metabolome Project library (http://www.hmdb.ca) and has been presented in previous studies (13,14). The ^1^H NMR spectrum can be identified in numerous endogenous products of the metabolism, including amino acids and fatty acids. These substances are involved in various biological processes, such as sugar, lipid and amino acid metabolism; therefore, the hydrogen spectrum diagram can be used as a chemical fingerprint spectrum to describe the extent of the changes in levels of endogenous products of metabolism in patients with COPD. According to the number of samples, |r|=0.273 was used as the cutoff value of discriminative significance at the level of P=0.05.

Typical ^1^H NMR spectra of the plasma from the patients with COPD and healthy controls are shown in Fig. 1. Resonance assignments were performed by The Metabolomics Innovation Center (http:/metabolomics.ca) and confirmed with the use of 2D NMR methods, such as COSY, TOCSY and J-Res spectra. The metabolites confirmed in the spectra of plasma included a number of amino acids, such as leucine, isoleucine, valine, alanine, citrulline, tyrosine, histidine and glutamine; a range of sugars, including α- and β-glucose; lipid metabolites, such as very low-density lipoprotein (VLDL), low-density lipoprotein (LDL) and unsaturated lipids; acidic metabolites, such as lactate, formate, acetate, acetoacetate, pyruvate, malonic acid and β-hydroxybutyrate; and other metabolites, such as glycoprotein, myo-inositol, scyllo-inositol, creatine, acetone and carnitine.

### OPLS-DA results

**Figure 2:**
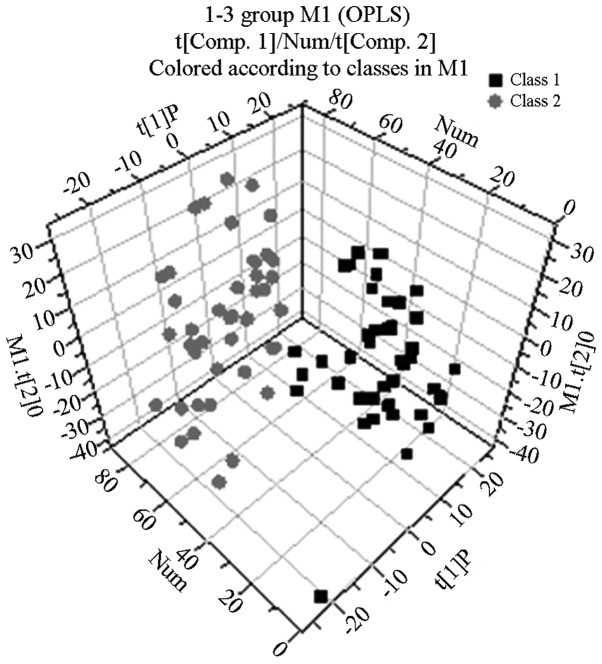
OPLS-DA scatter plot generated from ^1^H nuclear magnetic resonance spectra of plasma from patients with chronic obstructive pulmonary disease with abnormal Savda syndrome (gray circles) and healthy controls (black squares). The model parameters were R^2^X[1]=0.153587, R^2^X[2]=0.230561, R^2^Y=0.83 and Q^2^=0.68. OPLS-DA, orthogonal projection to latent structure-discriminate analysis. t[1]P, the principal component of the three-dimensional figure; Num, the percentage of the principal component.

**Figure 3:**
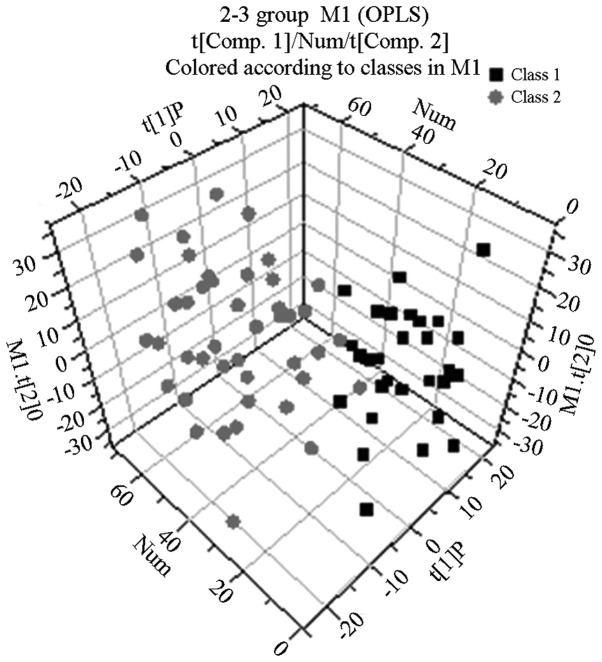
OPLS-DA scatter plot generated from ^1^H nuclear magnetic resonance spectra of plasma from healthy controls (black squares) and patients with chronic obstructive pulmonary disease with non-abnormal Savda syndrome (gray circles). The model parameters were R^2^X[1]=0.13, R^2^X[2]=0.25, R^2^Y=0.83 and Q^2^=0.67. OPLS-DA, orthogonal projection to latent structure-discriminate analysis. t[1]P, the principal component of the three-dimensional figure; Num, the percentage of the principal component.

**Figure 4:**
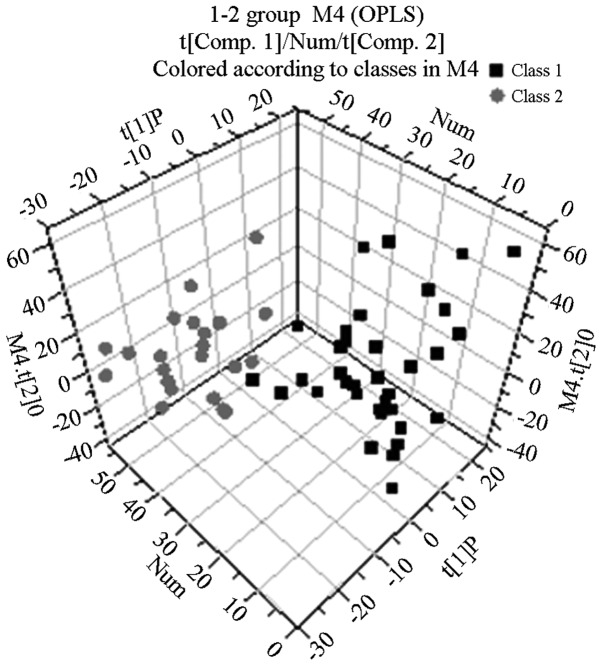
OPLS-DA scatter plot generated from ^1^H nuclear magnetic resonance spectra of plasma from patients with chronic obstructive pulmonary disease with abnormal Savda syndrome (gray circles) and patients with chronic obstructive pulmonary disease with non-abnormal Savda syndrome (black squares). The model parameters were R^2^X[1]=0.30, R^2^X[2]=0.275, R^2^Y=0.81 and Q^2^=0.61. OPLS-DA, orthogonal projection to latent structure-discriminate analysis. t[1]P, the principal component of the three-dimensional figure; Num, the percentage of the principal component.

Following the use of OPLS-DA to analyze the ^1^H NMR spectra integral values, the 3D spatial distribution was determined (Fig. 2–4). The regional distributions of the three groups were completely separate, as demonstrated by comparisons among the groups. In addition, notable differences were observed among the metabolic components present in the plasma from the three groups.

By analyzing the ^1^H NMR spectra and the 2D spectra obtained using the COSY, TOCSY and J-RES spectrum techniques, the signals belonging to the different plasma metabolic components in the normal control, abnormal Savda syndrome and non-abnormal Savda syndrome groups were determined.

### Western medicine diagnosis on COPD cases

#### General material distribution in COPD patients

A total of 103 cases of patients were examined. Among them there were 50 patients with severe COPD (level III), including 31 males and 19 females with an average age of 68.56 ± 12.451 years; there were also cases with moderate COPD (level II), including 25 males and 13 females at an average age of 58.727 ± 13.253 years. Finally, there were 15 patients with mild COPD (I level), including 9 males and 6 females, at an average age of 49.820 ± 14.278 years.

#### Distribution of patients with TUM sydrome

A total of 48 patients with abnormal Savda syndrome were examined. In total, 34 cases had severe COPD (level III), 11 cases had moderate COPD (level III) and 3 cases had mild COPD (level I). A total of 55 cases with normal Savda syndrome were examined. Amongst them, there were 16 cases with severe COPD (level II), 27 cases with moderate COPD (level II) and 12 cases with mild COPD (level I).

## Discussion

According to the TUM theory, patients with COPD can be classified according to whether they exhibit abnormal Savda syndrome or non-abnormal Savda syndrome. The main purpose of this study was to find biomarkers to analyze the characteristic mechanism of patients with COPD with abnormal Savda syndrome. The results showed that abnormal Savda syndrome was the predominant type of syndrome among patients with COPD; increased age, a longer duration of illness and a higher disease severity were characteristic of this type of syndrome compared with the non-abnormal Savda syndrome. This suggests that the appearance of the abnormal Savda syndrome in patients with COPD may take a longer time to develop in the course of disease. In addition, the OPLS-DA showed that the regional distributions of the COPD with abnormal Savda syndrome, COPD with non-abnormal Savda syndrome and healthy control groups were completely separate. Furthermore, compared with the healthy controls, the blood plasma of the patients with COPD with abnormal Savda syndrome exhibited a lower concentration of amino acids (including isoleucine, leucine, valine, phenylalanine, alanine, glutamine and tyrosine), glycoprotein and LDL, but a higher concentration of unsaturated lipids, lactic acid, carnitine, acetone and acetoacetic acid (Table I).

With the exception of lactic acid and phenylalanine, the metabolic components in the blood plasma of the patients with COPD with non-abnormal Savda syndrome differed from those in the plasma of the healthy controls (Fig. 3 and Table I). The OPLS-DA showed that the distribution areas of the metabolic components in the blood plasma of the patients with COPD and those with abnormal Savda syndrome and non-abnormal Savda syndrome could be distinguished (Fig 4). Two types of amino acids, namely alanine and isoleucine, were absent; however, there were another 13 types of metabolites of which the content was lower in the group of patients with COPD. Therefore, the differences of these 13 types of metabolites between the two groups of patients could be used as specific plasma biomarkers to identify abnormal Savda syndrome.

The present study analyzed the plasma metabolomic characteristics of patients with COPD with Uyghur syndrome (patients with abnormal and non-abnormal Savda syndrome). The blood plasma of patients with COPD exhibited a low concentration of amino acids, glycometabolism abnormalities, decreases in metabolites associated with immune function, including glutamine, and enhanced fat metabolism; signals for acetoacetic acid and acetone, which are the products of fatty acid catabolism, known as ketone bodies, were increased significantly in the plasma of patients with COPD compared with those in the plasma of healthy controls.

COPD patients with abnormal Savda syndrome rely mainly on energy from fatty acids. In the present study, such patients showed the most significant increases in ketone body acetoacetic acid (acetoacetate) and acetone content in the plasma (P<0.05). Ketone bodies are produced by the mitochondria in liver cells from fatty acids; a high content of blood ketone in the normal body is rare, but levels increase with an increased usage of fat. Furthermore, the content of carnitine from the fat metabolism increases accordingly (15).

Amino acids are the basic unit of protein composition, and the three branched chain amino acids (BCAAs), valine, leucine and isoleucine, are important and effective nutrition supplements. Numerous types of amino acid, including BCAAs, affect protein synthesis and decomposition (14), increase the body’s immune protection (16) and improve endurance in sports. Reduced production of these amino acids can cause fatigue; however, brain serotonin generation can reduce mental fatigue to help the organism naturally, without any side effects, thereby enhancing muscle activity or increasing energy, particularly at the cellular level, and reducing the characteristics of diseases, such as heart failure or chronic lung disease. COPD primarily affects the lungs, but the systemic inflammatory response, muscle malnutrition, languid performance and emaciation are associated with the various types of amino acid deficiency, including BCAA deficiency. In addition, the energy consumption associated with patients with COPD is enhanced, requiring an increase in the energy produced from fat metabolism.

In the present study, the blood plasma of patients with COPD exhibited significantly reduced lipoprotein and unsaturated lipid concentrations (P<0.01). We speculate that in patients with COPD with reduced pulmonary ventilation function there is an increased demand for energy metabolism; therefore, the catabolism of the energy-giving nutrients, carbohydrate, protein and fat, is increased as a compensatory mechanism. As a result, the carbohydrate, protein and fat resources are consumed at a higher rate, reducing the amino acid and fat content in the plasma. In order for the consumption of carbohydrate, protein and fat in the body to be increased, the metabolite content of plasma amino acids and lipids was significantly decreased in patients with COPD. A lack of protein may lead to a reduced synthesis of apolipoprotein and a reduction in the secretion of triglycerides into the blood following synthesis in liver cells. This may ultimately lead to a reduced fat metabolism.

Persistent COPD airway inflammation leads to smooth muscle cell proliferation, airway wall thickening and remodeling, airway mucosa congestion and edema, airway obstruction and respiratory muscle load increase. In contrast to skeletal muscle, respiratory muscle gains approximately half of its energy from sugar metabolism, while the other half is predominantly from lipid metabolism with fatty acids (17,18). In cases of severe COPD with abnormal Savda syndrome, the patients’ ability to breathe is poor, oxygen is limited, the aerobic oxidation pathway is restrained and the demand of the respiratory muscles for energy is increased. The supply of energy to the respiratory muscle either relies on glycolysis, which is low in efficiency and produces a large amount of lactic acid, which leads to a significant increase in the lactic acid (lactate) levels, or on energy from fatty acids. This study showed that the concentration of ketone bodies, such as acetoacetic acid (acetoacetate) and acetone, was increased significantly in the plasma of patients with COPD with abnormal Savda syndrome compared with that in the plasma from healthy controls (P<0.05). Ketone bodies are a type of metabolite that are catabolized from fatty acids in the mitochondria in liver cells; the concentration of blood ketone bodies in healthy individuals is low, and only increases naturally if the amount of fat utilized increases (18). The concentration of carnitine, which is associated with fat metabolism, was also increased.

It was also observed in this study that, in the plasma of patients with COPD with abnormal Savda syndrome, the concentration of other metabolites, such as glycoprotein, was lower than that in the plasma of patients with COPD with non-abnormal Savda syndrome and that in the plasma of healthy subjects. Certain types of plasma immune globulins belonging to the glycoprotein family exhibit efficacy in blood coagulation and antibody activity. Another important function of glycoprotein is a direct or indirect involvement in cell surface recognition, in order to protect the integrity of epithelial cells and increase the total number of lymphocytes to boost the function of the immune system (19). While the injury to the lung and airway epithelial cell integrity of patients with COPD with abnormal Savda syndrome is particularly serious, decreased immune function leads to the repeated aggravation of the illness.

COPD with abnormal Savda syndrome is the most serious syndrome of TUM, with persisting airway inflammation, irreversible airflow obstruction and an existing systemic inflammatory response, which results in the consumption of body energy and malnutrition. This leads to disordered energy metabolism, including the metabolism of amino acids, protein and fat. The pathological mechanism affects numerous parts of the body and multiple organs or organ systems and causes a loss of balance; thus, a vicious circle exists between diseases and disorders of overall function.

In conclusion, through metabolomic study, the specific metabolic markers reflecting the pathophysiological changes in patients with COPD with abnormal Savda syndrome were determined. The results of this study may enrich the theory of TUM and provide an objective view of the syndromes associated with TUM, thus enhancing their significance. When lesions are of the TUM abnormal Savda Syndrome type, targeted metabolomics can be performed with effective drug intervention targets, and an intervention with traditional medicine in advance may ultimately provide a good platform for improving the treatment of COPD and other complex diseases.

## Figures and Tables

**Table I:** Changes in metabolites observed in plasma obtained from the three groups and the correlation coefficients.

Metabolite	Chemical shift in ppm (multiplicity)	Assignment	Abnormal Savda syndrome/Healthy (r)	Non-abnormal Savda syndrome/Healthy (r)	Abnormal Savda syndrome/Non-abnormal Savda syndrome (r)
Lipid (VLDL)	0.85 (m)	CH_3_(CH_2_)_n_	0.81	0.72	0.66
	0.88 (m)	CH_3_CH_2_CH_2_C			
Isoleucine	0.93 (t)	δ-CH_3_	0.80	0.68	
	1.00 (d)	β-CH_3_			
	1.96 (m)	β-CH			
Leucine	0.95 (d)	δ-CH_3_	0.73	0.70	0.56
	0.97 (d)	δ-CH_3_			
	1.72 (m)	β-CH_2_/γ-CH			
	3.65 (dd)	α-CH			
Valine	0.98 (d)	CH_3_	0.77	0.71	0.63
	1.03 (d)	CH_3_			
	2.26 (d)	β-CH_2_			
	3.60 (d)	α-CH_2_			
Lactate	1.33 (d)	CH_3_	0.68		−0.48
	4.11 (q)	CH			
Alanine	1.47 (d)	CH_3_	0.71	0.69	
	3.76 (q)	α-CH			
Glycoprotein	2.03 (s)	NHCO-CH_3_	0.74	0.69	−0.49
Glutamate	2.13 (m)	half β-CH_2_	0.84	0.85	0.65
	2.36 (m)	half γ-CH_2_			
	3.75 (t)	α-CH			
Carnitine	2.22 (s)	CH_3_	−0.65	−0.57	−0.54
Tyrosine	2.52 (d)	half CH_2_	0.79	0.69	0.60
	2.67 (d)	half CH_2_			
Phenylalanine	3.03 (s)	CH_3_	0.78		0.61
	3.93 (s)	CH_2_			
Glutamine	2.10 (m)	half β-CH_2_	0.75	0.56	0.51
	2.14 (m)	half γ-CH_2_			
Unsaturated lipid	5.28 (m)	CHCH_2_CH_2_	0.78	0.76	0.36
	5.30 (m)	CH=CHCH_2_CH=CH			
Acetone	2.22 (s)	-CH_3_	−0.46	−0.30	−0.36
Acetoacetate	2.27 (s)	-CH_3_	−0.39	−0.31	−0.33

Multiplicity: s, singlet; d, doublet; t, triplet; q, quartet; m, multiplet; dd, doublet of doublets. VLDL, very low-density lipoprotein.
